# Exogenous hydrogen sulfide mitigates NLRP3 inflammasome-mediated inflammation through promoting autophagy via the AMPK-mTOR pathway

**DOI:** 10.1242/bio.043653

**Published:** 2019-07-17

**Authors:** Honggang Wang, Peiyu Zhong, Leilei Sun

**Affiliations:** Institute of Biomedical Informatics, Bioinformatics Center, School of Basic Medical Sciences, Henan University, Kaifeng 475000, China

**Keywords:** Hydrogen sulfide, Oleic acid, NLRP3 inflammasome, Autophagy, Inflammation

## Abstract

The aim of this study was to investigate whether exogenous hydrogen sulfide (H_2_S) could mitigate NLRP3 inflammasome-mediated inflammation through promoting autophagy via the AMPK-mTOR pathway in L02 cells. L02 cells were stimulated with different concentrations of oleic acid (OA), then cell viability and the protein expression of NLRP3 and pro-caspase-1 were detected by MTT and western blot, respectively, to determine appropriate OA concentration in this study. The cells were divided into four groups: the cells in the control group were cultured with RPMI-1640 for 24.5 h; the cells in the OA group were cultured with RPMI-1640 for 0.5 h, then were stimulated with 1.2 mmol/l OA for 24 h; the cells in the NaHS+OA group were pretreated with sodium hydrogen sulfide (NaHS, a donor of H_2_S) for 0.5 h before exposure to OA for 24 h; and the cells in the NaHS group were treated with NaHS 0.5 h, then were cultured with RPMI-1640 for 24 h. Subsequently, the cells in every group were collected and the protein expression of NLRP3, procaspase-1, cleaved caspase-1, P62, LC3, Beclin1, T-AMPK, P-AMPK, T-mTOR, P-mTOR and the level of IL-1β were detected by western blot and ElISA, respectively. Exogenous H_2_S reduced the level of NLRP3, caspase-1, P62, IL-1β and the ratio of P-mTOR/T-mTOR induced by OA and increased the ratio of LC3 II/I and the protein expression of Beclin1 suppressed by OA. This study demonstrates for the first time that H_2_S might suppress NLRP3 inflammasome-mediated inflammation induced by OA through promoting autophagy via the AMPK-mTOR pathway. It provides a theoretical basis for the further study of the anti-inflammatory mechanism of H_2_S.

## Abstract (precis)

**Summary:** We prove that exogenous H_2_S can suppress NLRP3-mediated inflammation by promoting autophagy via the AMPK/mTOR pathway in L02 cells, providing insights into the anti-inflammatory mechanism of H_2_S.

## INTRODUCTION

Inflammasomes were first proposed in 2002 as a multi-protein complex, which is a component of the organism immune system that recognizes pathogenic microorganisms and endogenous risk signals, namely pathogen-associated molecular patterns and damage-related molecular patterns, and other protein complexes. By activating caspase-1, the inflammasome induces the maturation and secretion of the pro-inflammatory factors interleukin-1 beta (IL-1β) and interleukin-18 (IL-18), regulates inflammatory response, resists pathogenic infection and stress injury and regulates caspase-1-dependent programmed cell death, but its over-activation can lead to tissue and organ inflammatory injury (Martinon, et al., 2002; Elliott and Sutterwala, 2015; Jo et al., 2016). Among inflammasomes, NOD-like receptor family 3 (NLRP3 inflammasome), composed of NLRP3, ASC and pro-caspase-1, is the most thoroughly studied inflammasome. It has been confirmed that NLRP3 inflammasome plays an important role in the pathogenesis and development of inflammation in many diseases (Jo et al., 2016). However, the exact regulatory mechanism has not been fully studied. In recent years, the relationship between NLRP3 inflammasome and autophagy has gradually become a research hotspot.

Autophagy is a process of self-sustaining internal environment stability in eukaryotic cells, in which pathogens, abnormal proteins and organelles are encapsulated by the bilayer membranes to form autophagosomes and then transferred to lysosomes for degredation (Sir et al., 2010; Qiu et al., 2014; Murrow and Debnath, 2013; Kimura, 2014). Autophagy can be classified into macroautophagy, microautophagy and chaperone-mediated autophagy based on the inducing signals, its timing, the types of targets and pathways of delivery of cargo into the lysosome (Gomes et al., 2017; Parzych and Klionsky, 2014). Among them, macroautophagy is the most studied autophagy, in which the content is wrapped by bilayer membrane structure to form autophagosome and then fuses with lysosome for degradation. Microautophagy refers to the lysosomal membrane directly invaginating and then encapsulating the cell contents. Chaperone-mediated autophagy is selective, in which molecular chaperones identify specific protein substrates with molecular chaperones and then fuse with lysosomes (Rubinsztein et al., 2012). Under physiological conditions, autophagy is often maintained at the basic level. The internal and external factors such as ischemia, hypoxia, pathogenic infection, hormone therapy, protein misfolding and nutritional deficiency can induce autophagy (Matsui et al., 2007). When the body is in the pathological state, the remarkably enhanced autophagy can remove the abnormal protein in the cell, which is beneficial to the survival of the cell. The effect of autophagy on the cell is a double-edged sword, since autophagy can cause autophagic death if the autophagy remains at a high level (Wu et al., 2019). It has been reported that autophagy is closely related to inflammation mediated by NLRP3 inflammasome. In microglia stimulated by PrP106-126, NLRP3 inflammasome negatively regulates autophagy (Lai et al., 2018). Enhanced autophagy can inhibit inflammation of macrophages and microglia mediated by NLRP3 inflammasome and alleviate pulmonary fibrosis by inhibiting NLRP3 inflammasome induced by AngII (Xue et al., 2019; Meng et al., 2019). At present, the relationship between autophagy and inflammation has not been fully studied.

Hydrogen sulfide (H_2_S), which is considered to be the third kind of gas signal molecule after CO and NO, is a colorless gas with an eggy odor. H_2_S was thought to be a toxic gas, until it was discovered to have many important functions and regulate many physiological and pathological processes in the 1990s. In the enzymatic pathway, H_2_S is produced by cystathionine beta synthase (CBS), cystathionine gamma lyase (CSE) and 3-mercaptopyruvate sulfotransferase (3-MST) (Kimura, 2014, 2015; Wang et al., 2013). In recent years, the anti-inflammatory effects of H_2_S have been gradually found. Exogenous H_2_S can significantly improve myocardial inflammatory injury induced by ischemia, reduce the release of inflammatory mediators, inhibit the production of inflammatory mediators in primary myocardial cells of rats induced by lipopolysaccharide (LPS) and alleviate inflammatory injury of gastric mucosal cells induced by ischemia-reperfusion (Sodha et al., 2009; Elrod et al., 2007; Toldo et al., 2014; Guo et al., 2014). Exogenous H_2_S also inhibits oxidative stress response and weakens LPS-induced acute renal inflammatory injury (Chen et al., 2018a,b), inhibits the activation of inflammasome by sodium urate crystallization and reduces the release of proinflammatory factors (Castelblanco et al., 2018). At present, the relationship between H_2_S and NLRP3 inflammasome has gradually become a research hotspot. Exogenous H_2_S can inhibit the expression of NLRP3 inflammasome induced by fatty acid and attenuate the cardiomyocytes injury induced by high glucose through inhibiting the activation of NLRP3 inflammasome (Luo et al., 2017; Huang et al., 2016). In addition, exogenous H_2_S can play a protective role by inhibiting autophagy, while enhanced autophagy can inhibit NLRP3 inflammasome (Yang et al., 2018; Ge et al., 2019). So far, the relationship among H_2_S, autophagy and NLRP3 inflammasome has not been studied by others. Our previous research showed that exogenous hydrogen sulfide mitigates LPS+ATP-induced inflammation by inhibiting NLRP3 inflammasome activation and promoting autophagy in L02 cells (Wu et al., 2019). However, the molecular mechanism of these effects has not been studied further. In this paper, we used OA to stimulate human hepatocyte L02 to establish an inflammatory model and sodium hydrogen sulfide (NaHS) to release exogenous H_2_S to verify the effect of H_2_S on NLRP3 inflammasome-mediated inflammation and study its mechanism to provide a theoretical basis for the further study of the anti-inflammatory mechanism of H_2_S and the development of anti-inflammatory drugs targeting NLRP3 inflammasome and autophagy.

## RESULTS

### OA induced NLRP3 inflammasome in L02 cells

**Fig. 1.**
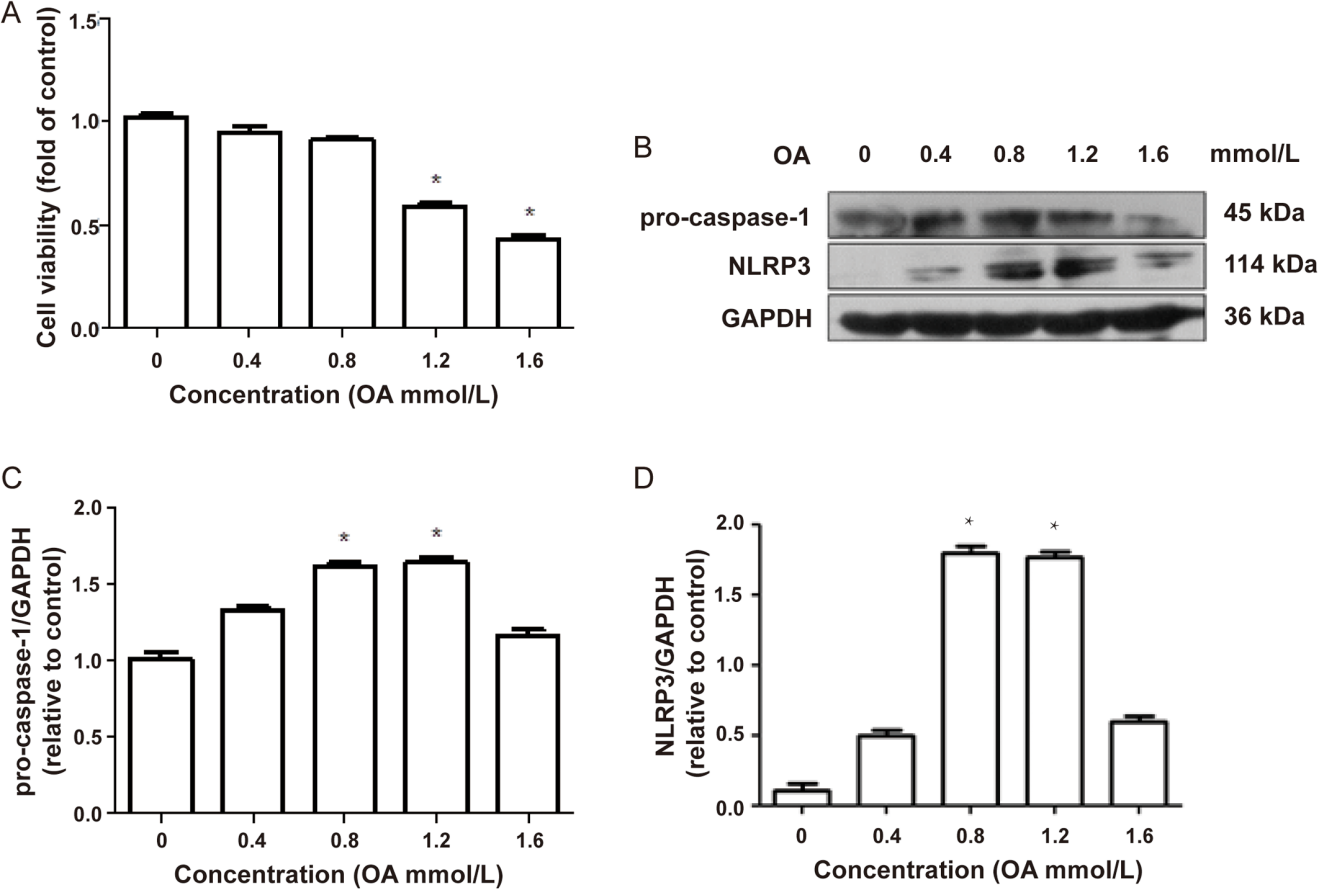
**OA-induced NLRP3 inflammasome in L02 cells.** (A) L02 cells were treated with OA at different concentrations (0, 0.4, 0.8, 1.2, 1.6 mmol/l) for 24 h, MTT was used for cell viability detection. (B–D) Western blot analysis was performed to assess the induction of NLRP3 and procaspase-1. Results are representative of three independent experiments. *P<0.05 versus control.

To determine the appropriate concentration of OA used to construct an inflammation model, we investigated the effect of different concentrations of OA on L02 cells. MTT analysis showed a notable decrease in the cell viability treated by 1.2 mmol/l and 1.6 mmol/l OA compared to the control group (Fig. 1A) and a marked increase of the expression of NLRP3 and procaspase-1 was observed in cells treated by 1.2 mmol/l OA compared to the control group (Fig. 1B–D). Based on the above results, 1.2 mmol/l OA was used for the subsequent establishment of inflammatory model.

### NLRP3-siRNA decreased the inflammation induced by OA

**Fig. 2.**
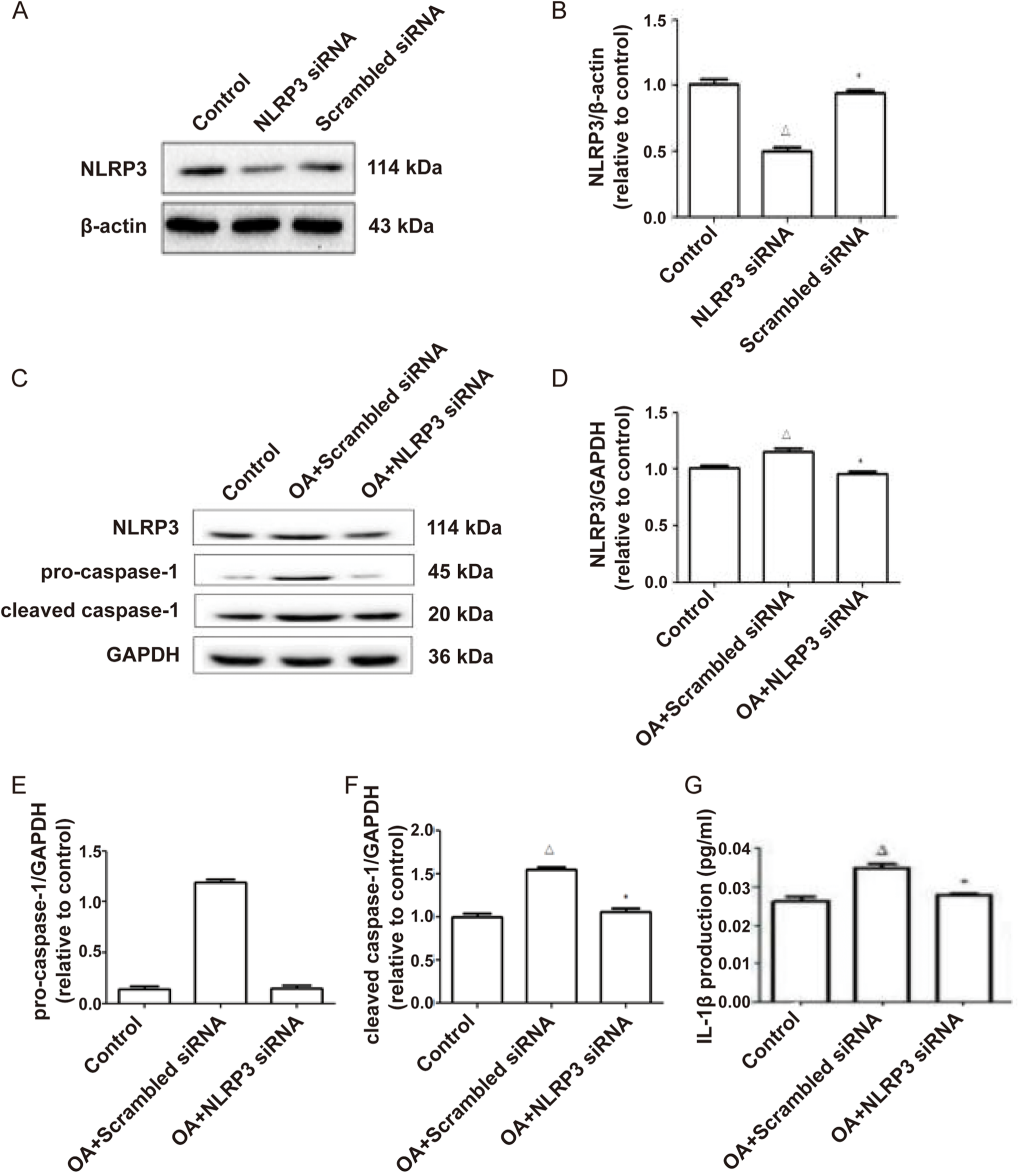
**NLRP3-siRNA decreases the protein expression of NLRP3 inflammasome and the inflammation induced by OA.** (A,B) L02 cells were transfected with either NLRP3-siRNA or scrambled siRNA. Western blot was performed to assess the protein expression of NLRP3. (C–F) L02 cells were then transfected with NLRP3-siRNA or scrambled siRNA followed by stimulation with OA and western blot was performed to assess the protein expression of NLRP3, procaspase-1 and cleaved caspase-1. (G) The IL-1β content in culture supernatant was detected by ELISA. Data from three independent experiments are presented as means±s.e.m. ^△^*P*<0.05 versus control. *P<0.05 versus OA+Scrambled siRNA group.

To investigate the role of NLRP3 in OA-induced inflammation, we used NLRP3-siRNA to inhibit NLRP3 to study whether OA induced inflammation through NLRP3. NLRP3-siRNA decreased the protein level of NLRP3 in L02 cells (Fig. 2A,B). Compared with the control group, the protein expression of NLRP3, pro-caspase-1 and cleaved caspase-1, and the IL-1β content in the culture supernatant in the OA+Scrambled siRNA group were increased. However, the protein expression, of NLRP3, pro-caspase-1 and cleaved caspase-1, and the IL-1β content in the culture supernatant in the OA+NLRP3-siRNA group were decreased compared with the OA+scrambled siRNA group (Fig. 2C–G), which suggested that NLRP3 mediated OA-induced inflammation.

### Exogenous H_2_S attenuated NLRP3 inflammasome and the inflammation induced by OA

**Fig. 3.**
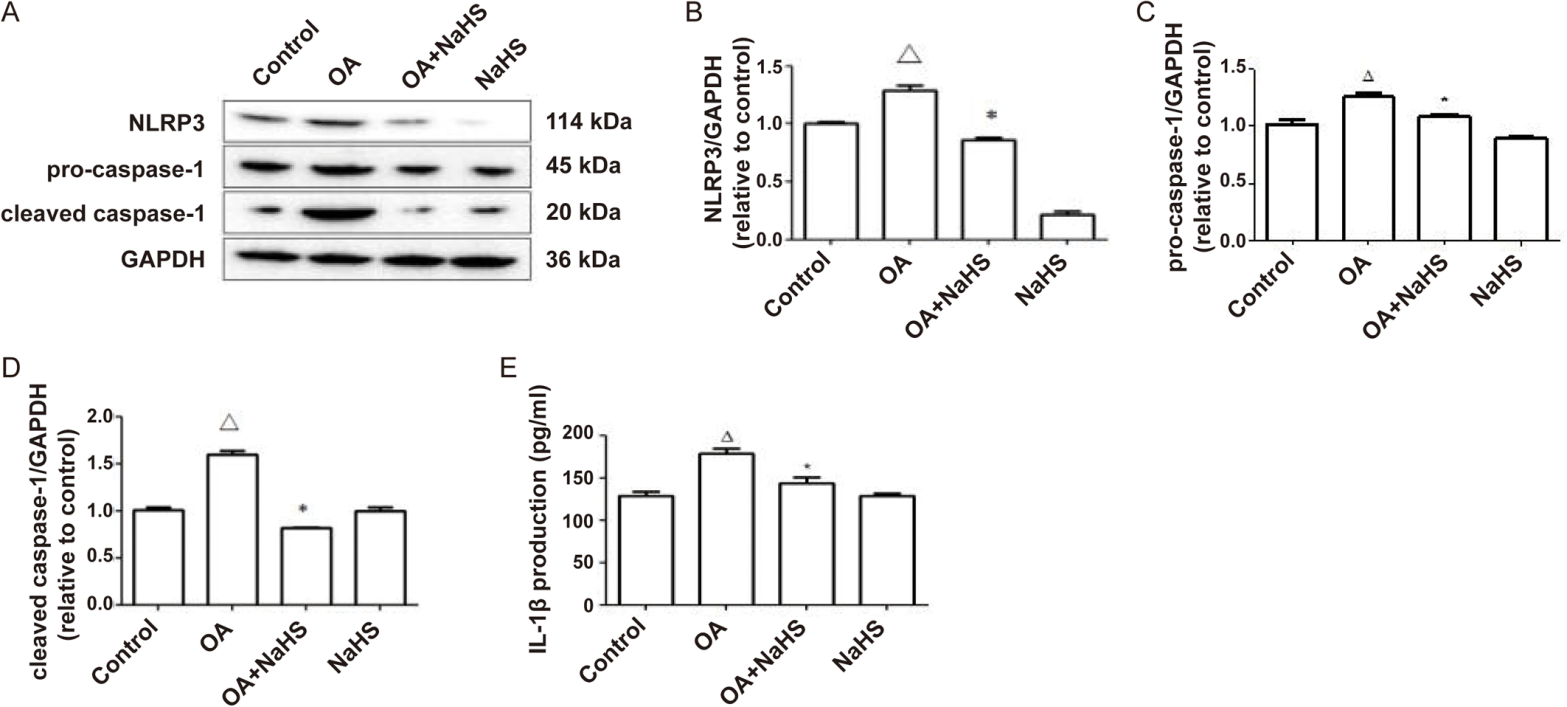
**Exogenous H_2_S attenuated the protein expression of NLRP3 inflammasome and the inflammation induced by OA in L02 cells.** (A–D) L02 cells were pretreated with NaHS 0.5 h before exposure to OA for 24 h. Western blot was performed to assess the protein expression of NLRP3, procaspase-1 and cleaved caspase-1. (E) The IL-1β content in culture supernatant was detected by ELISA. Results are representative of three independent experiments. ^△^*P*<0.05 versus control, *P<0.05 versus OA group.

We investigated the effect of H_2_S on the protein expression of NLRP3 inflammasome and the inflammation induced by OA. Compared with the OA group, the protein expression of NLRP3, procaspase-1 and cleaved caspase-1 in the OA+NaHS group were notably decreased (Fig. 3A–D) and the IL-1β content in the culture supernatant was also decreased (Fig. 3E), suggesting that exogenous H_2_S could inhibit the protein expression of NLRP3 inflammasome and the inflammation induced by OA.

### Exogenous H_2_S upregulated OA-suppressed autophagy

**Fig. 4.**
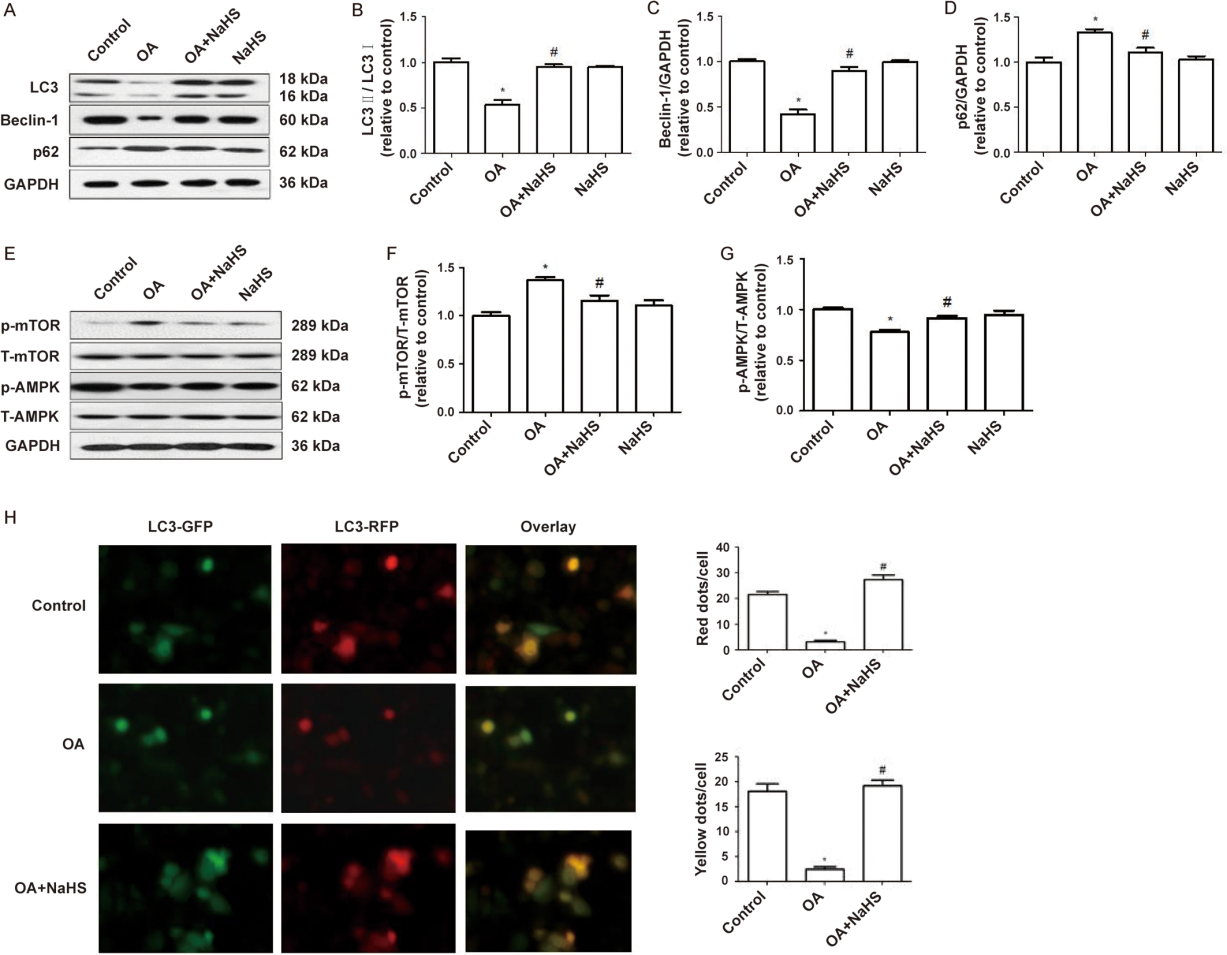
**Exogenous H_2_S upregulated OA-suppressed autophagy.** (A–G) L02 cells were pretreated with NaHS 0.5 h before exposure to OA for 24 h. Western blot was performed to assess the protein expression of LC3, Beclin-1, p62, P-AMPK, T-AMPK, P-mTOR and T-mTOR. The blots were stripped and re-probed with GAPDH as a loading control. Results is representative of three independent experiments. (H) RFP/GFP-tagged LC3 plasmids were transfected into L02 cells. Cells were pretreated with NaHS 0.5 h before exposure to OA for 24 h. Quantification and representative images of early autophagosomes (yellow puncta generated by overlapping of GFP plus RFP puncta) are shown as a yellow signal and late autolysosomes (red puncta) as a red signal. Scale bar: 200 μm. Results are representative of three independent experiments. *P<0.05 versus control, ^#^*P*<0.05 versus OA group.

Next, we investigated the effect of exogenous H_2_S on autophagy. The protein expression ratio of LC3II/I and the protein expression of Beclin-1 and p62 were indicative of autophagy level (Wu et al., 2019). The result of western blot showed that compared with the control group, OA decreased the protein expression ratio of LC3II/I and the protein expression of Beclin-1 and increased the protein expression of p62, which indicated that autophagy was suppressed by OA. Compared with the OA group, exogenous H_2_S increased the protein expression ratio of LC3II/I and the protein expression of Beclin-1 and decreased the protein expression of p62, which indicated that autophagy was promoted by exogenous H_2_S (Fig. 4A–D). In addition, we used GFP-tagged LC3 to detect autophagosomes and RFP-tagged LC3 to detect both autophagosomes and autolysosomes in L02 cells. In the merged images, the overlap of red dots and green dots are shown as yellow dots, which represent autophagosomes, while the other red dots represent the late autolysosomes. Treatment with OA reduced both autophagosomes (yellow dots) and autolysosomes (red dots) formation, and this effect was reversed by co-administration of NaHS (Fig. 4H). Collectively, H_2_S promoted OA-suppressed autophagy. We also detected the protein expression of signal molecules AMPK and mTOR in the AMPK/mTOR pathway and the results showed that the protein expression ratio of P-AMPK/T-AMPK in the OA+NaHS group was increased, while the protein expression ratio of P-mTOR/T-mTOR was decreased compared with the OA group, which indicated that the AMPK/mTOR pathway was promoted by exogenous H_2_S (Fig. 4E–G).

### Autophagy mediated the effect of H_2_S on NLRP3 inflammasome and inflammation induced by OA

**Fig. 5.**
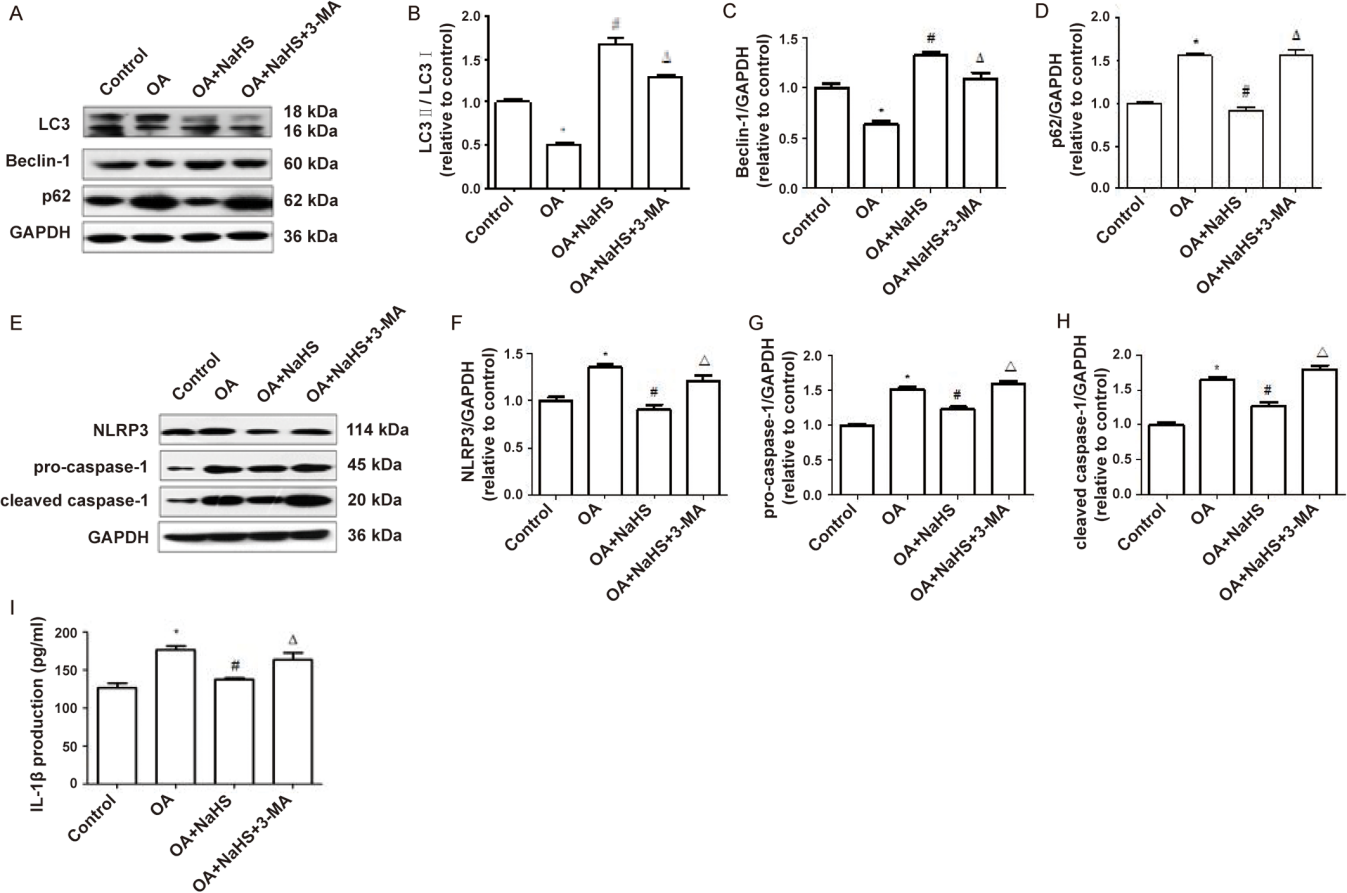
**3-MA suppressed autophagy and upregulated the protein expression of NLRP3 inflammasome and the inflammation suppressed by NaHS.** (A–I) L02 cells were pretreated with NaHS or 3-MA+NaHS before exposure to OA for 24 h. Western blot was performed to assess the protein expression of LC3, Beclin-1, p62, NLRP3, pro-caspase-1 and cleaved caspase-1. The blots were stripped and re-probed with GAPDH as a loading control. Results representative of three independent experiments. *P<0.05 versus control, ^#^*P*<0.05 versus OA group, ^△^*P*<0.05 versus OA+NaHS group.

To investigate whether exogenous H_2_S attenuated NLRP3 inflammasome and inflammation induced by OA through promoting autophagy, we used 3-Methyladenine (3-MA), an autophagy inhibitor, to suppress autophagy and the results showed that the protein expression ratio of LC3II/I and the protein expression of Beclin-1 in the OA+NaHS+3-MA group decreased significantly compared with the OA+NaHS group. The protein expression of p62 in the OA+NaHS+3-MA group increased significantly compared with the OA+NaHS group (Fig. 5A–D). These results indicate that 3-MA inhibited autophagy. The protein expression of NLRP3, procaspase-1 and cleaved caspase-1 in the OA+NaHS+3-MA group increased significantly compared with the OA+NaHS group (Fig. 5E–H). The IL-1β content in the culture supernatant of the OA+NaHS+3-MA group increased significantly compared with the OA+NaHS group (Fig. 5I). The above results indicate that when the autophagy is inhibited, the anti-inflammatory effects of H_2_S are also suppressed. We could deduce that exogenous H_2_S attenuated NLRP3 inflammasome-mediated inflammation induced by OA through promoting autophagy. In addition, the ratio of P-AMPK/T-AMPK in the OA+NaHS+3-MA group was decreased and the ratio of P-mTOR/T-mTOR was increased compared with the OA+NaHS group (Fig. S1).

### Exogenous H_2_S mitigates OA-induced NLRP3 inflammasome and inflammation through the AMPK/mTOR pathway

**Fig. 6.**
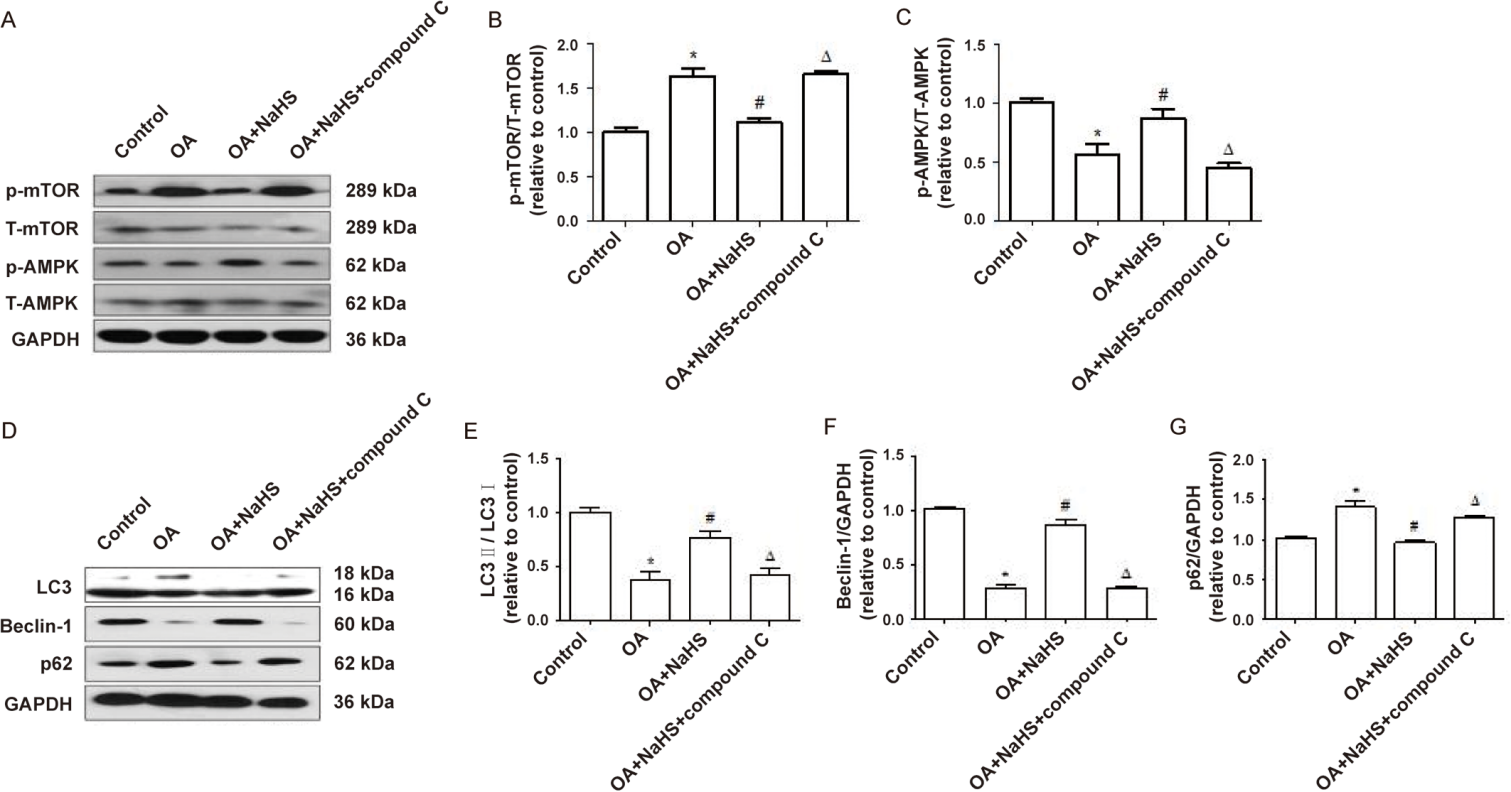
**Compound c suppressed AMPK/mTOR pathway and autophagy.** (A–G) L02 cells were pretreated with compound c or compound c+NaHS before exposure to OA for 24 h. Western blot was performed to assess the expression of P-AMPK, T-AMPK, P-mTOR and T-mTOR (A–C), and LC3, Beclin-1 and p62 (D–G). The blots were stripped and re-probed with GAPDH as a loading control. Results are representative of three independent experiments. *P<0.05 versus control, ^#^*P*<0.05 versus OA group, ^△^*P*<0.05 versus OA+NaHS group.

**Fig. 7.**
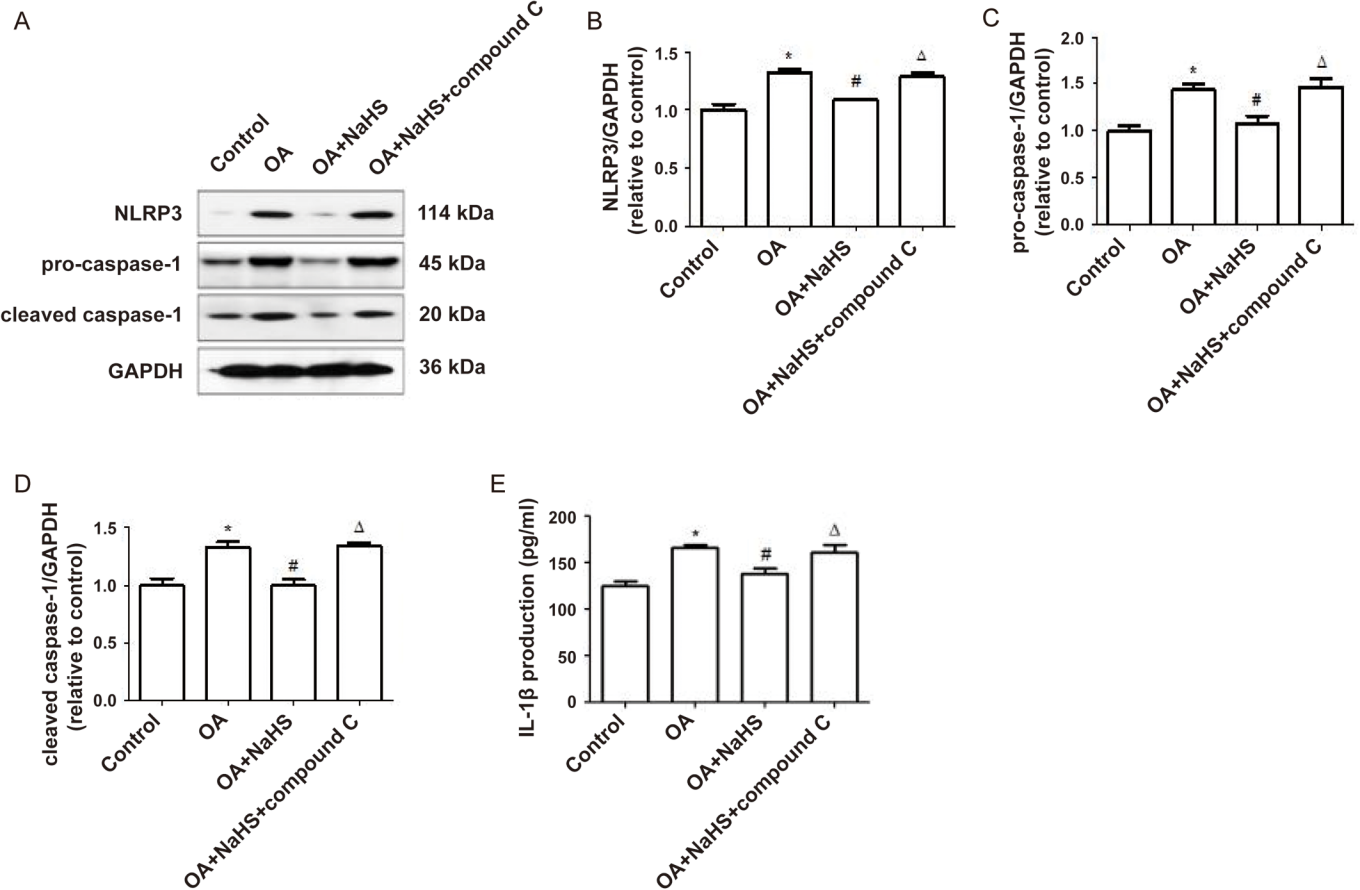
**Compound c activated H_2_S-suppressed NLRP3 inflammasome and inflammation.** (A–D) L02 cells were pretreated with compound c or compound c+NaHS before exposure to OA for 24 h. Western blot was performed to assess the expression of NLRP3, procaspase-1 and cleaved caspase-1. (E) The IL-1β content in culture supernatant was detected by ELISA. The blots were stripped and re-probed with GAPDH as a loading control. Results are representative of three independent experiments. *P<0.05 versus control, ^#^*P*<0.05 versus OA group, ^△^*P*<0.05 versus OA+NaHS group.

Finally, we investigated through which signaling pathway H_2_S promoted autophagy to play an anti-inflammatory role. The results showed that compared with the OA+NaHS group, compound c, an inhibitor of AMPK, decreased the protein expression ratio of P-AMPK/T-AMPK and LC3II/I (Fig. 6A,C–E), and the protein expression of Beclin-1 (Fig. 6D,F), but increased the protein expression ratio of P-mTOR/T-mTOR (Fig. 6A,B) and the protein expression of p62 (Fig. 6D,G), which suggests that H_2_S-promoted autophagy and the AMPK/mTOR pathway were suppressed by compound c. In addition, compared with the OA+NaHS group, compound c increased the expression of NLRP3, procaspase-1, cleaved caspase-1(Fig. 7 A–D) and the IL-1β content in the culture supernatant suppressed by H_2_S (Fig. 7E). The above results indicate that H_2_S mitigates NLRP3 inflammasome-mediated inflammation induced by OA through the AMPK/mTOR pathway.

## DISCUSSION

**Fig. 8.**
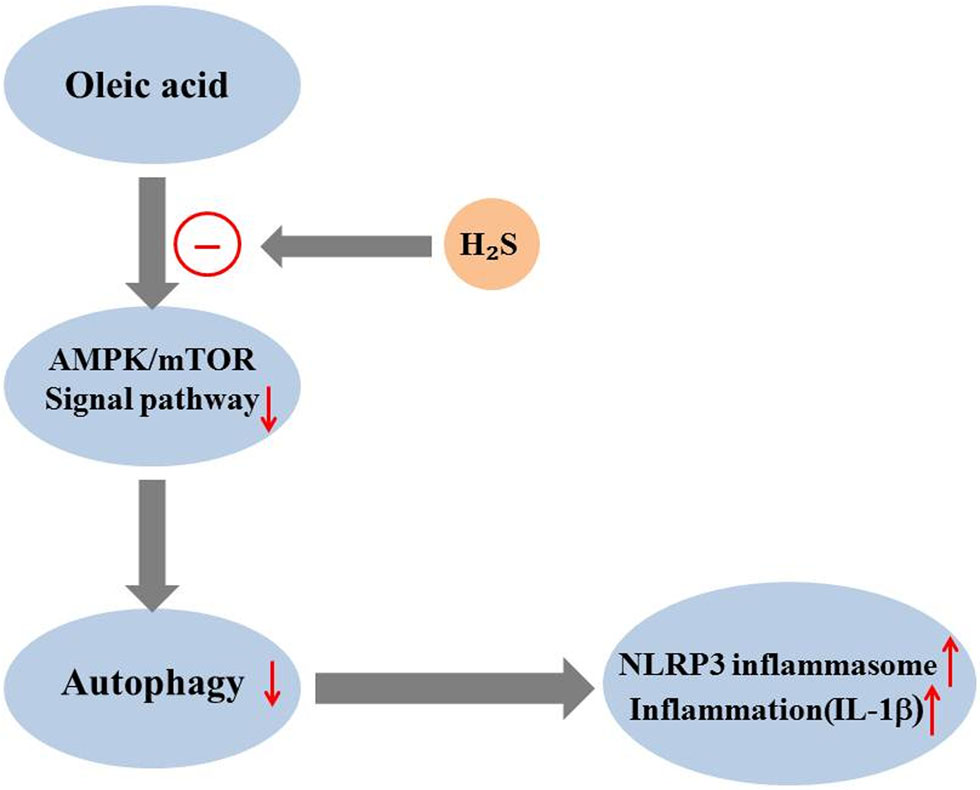
**Pathway of the protective effect of H_2_S on OA-induced NLRP3 inflammasome and inflammation in L02 cells.** OA suppressed the AMPK/mTOR signal pathway, then inhibited autophagy, and induced NLRP3 inflammasome and inflammation. The above effect of OA was counteracted by coadministration of exogenous H_2_S.

In the present study, we investigated whether exogenous H_2_S could mitigate NLRP3 inflammasome and inflammation induced by OA and its mechanism. The results show that in L02 cells: (1) exogenous H_2_S suppresses OA-induced inflammation and NLRP3 inflammasome; (2) exogenous H_2_S could promote autophagy suppressed by OA; (3) autophagy mediates the effect of H_2_S on NLRP3 inflammasome and inflammation induced by OA; and (4) exogenous H_2_S mitigates NLRP3 inflammasome-mediated inflammation induced by OA by promoting autophagy via the AMPK-mTOR pathway (Fig. 8).

Fatty acids can induce inflammation. Tadayoshi Karasawa et al. proved that excessive saturated fatty acids could lead to intracellular crystallization, then inhibit lysosome function, and ultimately activate NLRP3 inflammasome (Karasawa et al., 2018). In this paper, MTT results showed that 0.4 and 0.8 mmol/l of OA had no obvious cytotoxicity, but 1.2 mmol/l and 1.6 mmol/l of OA could significantly inhibit cell viability. Considering the results of western blot and MTT, we determined that the concentration of OA used in the follow-up experiments was 1.2 mmol/l which showed obvious pro-inflammatory and high cell viability.

H_2_S has been reported to protect cells from injury by inhibiting inflammation. Exogenous H_2_S can alleviate cardiac injury during acute myocardial ischemia by reducing inflammatory reaction in heart tissues under oxidative stress (Bai et al., 2018) and ameliorate LPS-induced diaphragm dysfunction in rats by reducing inflammation through ROS/MAPK and TLR4/NF-κB signaling pathways (Zhang et al., 2018). H_2_S also can mitigate adenine-induced chronic renal failure in rats by inhibiting inflammation through ROS/AMPK and nuclear factor-kappa B signaling pathways (Wu et al., 2017). In this study, we demonstrated that treatment with NaHS decreased the protein expression of NLRP3, pro-caspase-1, cleaved caspase-1 and the secretion of IL-1β, which suggests that exogenous H_2_S could mitigate OA-induced inflammation in L02 cells. Our findings were helpful to introduce H_2_S-related drugs into the clinical treatment of hepatitis.

We further investigated the role of NLRP3 in OA-induced inflammation in L02 cells. It has been reported that free fatty acids induced inflammation in cardiomyocyte or high glucose-induced inflammation in macrophages by activating NLRP3 inflammasome (Luo et al., 2017; Huang et al., 2016). Our results show that treatment with NLRP3-siRNA significantly decreases OA-induced NLRP3 inflammasome and the secretion of IL-β, indicating that OA induces inflammation via activating NLRP3 inflammasome.

In recent years, it has been found that autophagy is closely related to the protective effect of H_2_S. Pretreatment with NaHS could alleviate acute myocardial ischemia injury by suppressing autophagy under oxidative stress, significantly reducing brain damage and preserving the blood-brain barrier integrity after traumatic brain injury by inhibiting autophagy via activation of the PI3K/Akt/mTOR signaling pathway and mitigating acrylonitrile-induced decrease of cell viability through influencing autophagy (Bai et al., 2018; Xu et al., 2018; Yang et al., 2018). Exogenous H_2_S also might protect mouse kidney against unilateral ureteral obstruction by suppressing ROS-AMPK-mediated autophagy, ameliorating blood-spinal cord barrier disruption and improving functional recovery by suppressing endoplasmic reticulum stress-mediated autophagy and inducing the apoptosis of hepatocellular carcinoma cells through promoting autophagy via the PI3K/AKT/mTOR signaling pathway (Chen et al., 2018a,b; Wang et al., 2017, 2018). Autophagy has also been reported to be closely related to NLRP3 inflammasome-mediated inflammation. Autophagy ameliorated pulmonary fibrosis through suppressing NLRP3 inflammasome activation induced by ROS via redox balance modulation (Meng et al., 2019). In this study, we introduced autophagy to the anti-inflammatory effects of H_2_S and the results show that NaHS could increase the ratio of LC3-II/I and the protein expression of Beclin-1 and decrease the protein expression of p62, which indicates that OA-suppressed autophagy was activated by exogenous H_2_S, while 3-MA could counteract the activation of H_2_S. Moreover, 3-MA further enhanced H_2_S-suppressed NLRP3 inflammasome and IL-1β secretion induced by OA. The above results indicate that exogenous H_2_S could mitigate NLRP3-mediated inflammation induced by OA via promoting autophagy in L02 cells. Contrary to the promotion of autophagy, H_2_S can also protect cells by inhibiting autophagy. Exogenous H_2_S inhibited the apoptosis of retinal ganglion cells through downregulating autophagy and attenuated cerebral ischemia/reperfusion injury via suppressing overactivated autophagy in rats (Huang et al., 2018; Jiang et al., 2017). Autophagy had been considered as a double-edged sword that has the potential to promote survival or death in cerebral ischemia/reperfusion injury (Wei et al., 2012). In general, low levels of autophagy can protect cells, whereas sustained high levels of autophagy has the opposite effect. H_2_S protects cells by activating or inhibiting autophagy, depending on the type of tissue and the basic level of autophagy.

H_2_S could affect autophagy through multiple signaling pathways. It has been reported that H_2_S could reduce serum TG levels and ameliorate non-alcoholic fatty liver disease by activating autophagy via the AMPK-mTOR pathway (Sun et al., 2015). During myocardial ischemic reperfusion injury in neonatal rats, exogenous H_2_S activated the PI3K/SGK1/GSK3β signaling pathway to inhibit autophagy to reduce myocardial injury (Jiang et al., 2016). Our results show that NaHS could increase the ratio of P-AMPK/T-AMPK and decrease the ratio of P-mTOR/T-mTOR, while compound c, an inhibitor of AMPK, could suppress H_2_S-activated autophagy and enhance H_2_S-suppressed NLRP3 inflammasome and IL-1β secretion, which indicates that exogenous H_2_S could mitigate NLRP3-mediated inflammation induced by OA by promoting autophagy via the AMPK/mTOR signaling pathway in L02 cells. It could be inferred from the present results that the inhibition of the AMPK/mTOR signaling pathway may be helpful in the treatment of hepatitis.

This study is just the beginning of a series of studies; the mechanism of H_2_S affecting autophagy remains to be further studied. For example, which stage of autophagy does H_2_S influence to inhibit inflammation and can H_2_S regulate the fusion process of autophagy and lysosomes?

Saturated fatty acids are a good tool for establishing a cellular inflammation model (Legrand-Poels et al., 2014). In this study, we used a high concentration of OA, an unsaturated fatty acid, to stimulate cells to establish inflammation models., which is the deficiency of this article.

In conclusion, this study proves that exogenous H_2_S could suppress NLRP3-mediated inflammation by promoting autophagy via the AMPK/mTOR pathway in L02 cells. It provides insights into the anti-inflammatory mechanism of H_2_S and the novel H_2_S-related anti-inflammatory drugs targeting NLRP3 inflammasome, which can be designed and applied for the treatment of hepatitis in the future.

## MATERIALS AND METHODS

### Reagents

Sodium hydrogen sulfide (NaHS, #161527) was purchased from Sigma-Aldrich. The normal human hepatocytes L02 cells (#CL-0111) were purchased from Procell. The content of IL-1β was determined using a commercial ELISA kit (Elabscience, #E-EL-H0149c). The primary antibodies, including rabbit anti-NLRP3 (#Ag26289, 1:500), rabbit anti-LC3 (#14600-1-AP, 1:1000) mouse anti-AMPK (#10929-2-AP, 1:1000) and rabbit anti-mTOR (#28273-1-AP, 1:1000) were purchased from Proteintech, rabbit anti-cleaved caspase-1 (#89332, 1:1000), rabbit anti-procaspase-1 (#24232, 1:1000), rabbit anti-Beclin-1 (#3495, 1:1000), mouse anti-β-actin (#3700, 1:2000) and rabbit anti-GAPDH (#8884, 1:2000) were purchased from Cell Signaling Technology.

### L02 cell culture

L02 cells were cultured in RPMI-1640 with 10% fetal serum and penicillin (100 U/ml)/streptomycin (100 µg/ml) at 37°C under an atmosphere of 5% CO2.

### Cell grouping and treatment

L02 cells were divided into four groups: the cells in the control group were cultured with RPMI-1640 for 24.5 h; the cells in the OA group were cultured with RPMI-1640 for 0.5 h, then were stimulated with 1.2 mmol/l OA for 24 h; the cells in the NaHS+OA group were pretreated with NaHS (a donor of H_2_S) 0.5 h before exposure to OA for 24 h; and the cells in the NaHS group were treated with NaHS for 0.5 h, then were cultured with RPMI-1640 for 24 h.

To determine whether NLRP3 regulated OA-induced inflammation, the L02 cells were divided into three groups: the cells in the control group were cultured with RPMI-1640 for 48 h; the cells in the OA+Scrambled siRNA group were were transfected with Scrambled siRNA for 24 h followed by stimulation with 1.2 mmol/l OA for 24 h; the cells in the OA+siRNA-NLRP3 group were transfected with siRNA-NLRP3 for 24 h followed by stimulation with 1.2 mmol/l OA for 24 h.

### Cell viability assay

Cell viability was quantitated using 3-(4,5-dimethylthiazol-2-yl)-2,5-diphenyltetrazolium bromide (MTT) assay. L02 cells were grown in RPMI-1640 with 10% fetal bovine serum. Cells were plated in each well of a 96-well plate at 1×10^4^ cells/well, grown at 37°C under 5% CO_2_ atmosphere for 24 h, then 20 μl of MTT was added into each well, respectively. After being cultured for 4 h continuously, the culture medium was discarded and 150 μl of DMSO was added, then the 96-well plate was vibrated for 5 min, the absorbance value of 570 nm wavelength was tested three times.

### Western blot

After the culture solution was discarded, the cells in the six-well-plate were washed with cold PBS three times and 200 μl of RIPA lysate was added into each well. After being blended at room temperature for 10 min, the cells were transferred into a 1.5 ml centrifuge tube and lysed on the ice. After being centrifuged for 10 min at 12,000×***g***, 4°C, the supernatants were transferred into another 1.5 ml centrifuge tube. BCA kit was used to detect the protein concentration. SDS-PAGE electrophoresis were performed on the protein samples. The gel was soaked in the transfer buffer for 10 min. The specific primary antibodies including anti-NLRP3, anti-procaspase-1, anti-cleaved caspase-1, anti-cleaved caspase-3 and anti-GAPDH were added respectively [diluted with TBST containing 1% (w/v) skimmed milk power]. It was incubated at the room temperature for 2 h and then the membrane was washed with TBST three times, 5–10 min each time. Then the membrane was incubated with the secondary antibody (1:10,000). The membrane was washed with TBST three times, 5–10 min each time. The blot bands were visualized using an enhanced chemiluminescence system (Thermo Fisher Scientific). The bands were semi-quantified with ImageJ v2.1 analysis software.

### ELISA for detection of IL-1β in the culture supernatant

The concentrations of IL-1β in the culture supernatant were determined using enzyme-linked immunosorbent assay (ELISA) kits according to the manufacturer's instructions (Elabscience, Wuhan, China). The experiments were performed three times.

### Immunofluorescence

Immunofluorescence was performed according to routine protocols. To access autophagic flux, the tandem green fluorescent protein/red fluorescent protein (GFP/RFP)-tagged LC3 plasmid was transfected into L02 cells with Lipofectamine 2000 (Invitrogen, #11668027). The transfected cells were selected with G418 (400 nM; Roche, Basel, Swizerland) and then treated with OA or OA+NaHS. Autophagy was assessed by colocalization of the transgenes using ZEN software (Carl Zeiss, Jena, Germany) (Kimura et al., 2007).

### RNA interference and transfection

When L02 cells had grown to about 75% confluence, the siRNA targeting NLRP3 (Gene Pharma, Shanghai, China, sense, 5′-GCUUCAGCCACAUGACUUUTT-3′, and antisense, 5′-AGUCAUGUGGCUGAAGCTT-3′) or negative control siRNA (sense, 5′-UUCUCCGAACGUGUCACGUTT-3′, and antisense, 5′-ACGUGACAC GUUCGGAGAATT-3′) were transfected into cells with Lipofectamine transfection regent (Life Technologies, Carlsbad, USA). siRNA was dissolved in serum-free medium to a final concentration of 20 μM, then 5 μl siRNA and 5 μl Lipofectamine were mixed in a 500 μl buffer system at room temperature for 30 min to form siRNA/Lipofectamine complex, which were equally added into the wells of the six-well plate at 37°C in a 5% CO_2_ incubator. After being cultured for 24 h, cells were collected for analysis.

### Statistical analysis

The results were presented as the mean±s.e.m. (*n*=3). The *t*-test was employed for the comparison between two groups and one-way analysis of variance (ANOVA) was used to analyze the differences among multiple groups. A difference was considered significant at *P*<0.05.

## Supplementary Material

Supplementary information
