# Supplementary material for: Exogenous hydrogen sulfide mitigates NLRP3 inflammasome-mediated inflammation through promoting autophagy via the AMPK-mTOR pathway
Source: Biol Open. 2019 Jul 17;8(7):bio043653. doi: 10.1242/bio.043653 (PMC6679392; doi:10.1242/bio.043653)
Supplement: Supplementary information [file biolopen-8-043653-s1.pdf]

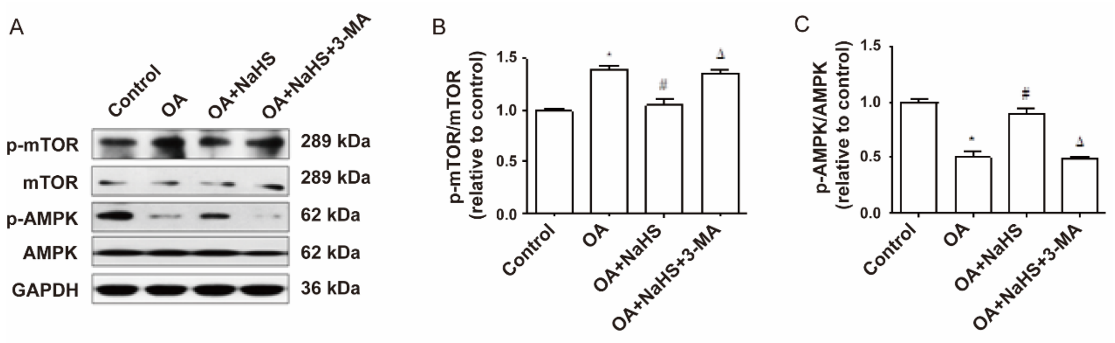

Figure S1. 3-MA suppressed the the protein expression of AMPK and mTOR

L02 cells were pretreated with NaHS or 3-MA+NaHS before exposure to OA for 24 h. Western blot were performed to assess the protein expression of P-AMPK, T-AMPK, P-mTOR, and T-mTOR. The blots were stripped and re-probed with GAPDH as a loading control. Experiment is representative of three independent experiments.$^{*}P<0.05$ vs. control, $^{\#}P<0.05$ vs. OA group, $^{\triangle}P<0.05$ vs. OA+NaHS group.
